# Alterations in multidimensional motor unit number index of hand muscles after incomplete cervical spinal cord injury

**DOI:** 10.3389/fnhum.2015.00238

**Published:** 2015-05-08

**Authors:** Le Li, Xiaoyan Li, Jie Liu, Ping Zhou

**Affiliations:** ^1^Department of Rehabilitation Medicine, The First Affiliated Hospital, Sun Yat-Sen UniversityGuangzhou, China; ^2^Department of Physical Medicine and Rehabilitation, University of Texas Health Science Center at Houston, and TIRR Memorial Hermann Research CenterHouston, TX, USA; ^3^Sensory Motor Performance Program, Rehabilitation Institute of ChicagoChicago, IL, USA; ^4^Biomedical Engineering Program, University of Science and Technology of ChinaHefei, China

**Keywords:** EMG, MD-MUNIX, MD-MUSIX, CMAP, SCI, hand muscles

## Abstract

The objective of this study was to apply a novel multidimensional motor unit number index (MD-MUNIX) technique to examine hand muscles in patients with incomplete cervical spinal cord injury (SCI). The MD-MUNIX was estimated from the compound muscle action potential (CMAP) and different levels of surface interference pattern electromyogram (EMG) at multiple directions of voluntary isometric muscle contraction. The MD-MUNIX was applied in the first dorsal interosseous (FDI), thenar and hypothenar muscles of SCI (*n* = 12) and healthy control (*n* = 12) subjects. The results showed that the SCI subjects had significantly smaller CMAP and MD-MUNIX in all the three examined muscles, compared to those derived from the healthy control subjects. The multidimensional motor unit size index (MD-MUSIX) demonstrated significantly larger values for the FDI and hypothenar muscles in SCI subjects than those from healthy control subjects, whereas the MD-MUSIX enlargement was marginally significant for the thenar muscles. The findings from the MD-MUNIX analyses provide an evidence of motor unit loss in hand muscles of cervical SCI patients, contributing to hand function deterioration.

## Introduction

Spinal cord injury (SCI) leads to various deficits in muscular function and affects the quality of daily life (Welch et al., 1986; Curt and Dietz, 1999). Muscle weakness, lower muscle contractile speed, fatigability of force gradation and loss of precision are common in hand muscles after cervical SCI and collectively deteriorate hand function (Farmer et al., 1993; Thomas, 1997; Thomas et al., 1997; Zijdewind and Thomas, 2012). These symptoms could be the results of different mechanisms, including weaker synchrony of motor unit discharge, reduced maximum motor unit firing rate, trans-synaptic motor neuron degeneration, loss of central motor neuron trophic influences, etc. (see review Thomas et al., 2014).

In order to design and optimize rehabilitation intervention after SCI, it is necessary to examine how the neurological deficits affect muscle control and force generation. Motor unit (which contains an alpha motor neuron, its axon and innervated muscle fibers) provides a fundamental structural and functional framework for such examinations (Smith et al., 1999). For example, it has been an extensive research topic for decades to explore the altered descending, spinal and afferent input, and intrinsic properties of spinal motor neurons after SCI (Kaelan et al., 1988). Different motor unit number estimation (MUNE) methods have also been used to investigate changes in spinal motor neuron or motor unit population after SCI (Yang et al., 1990; Xiong et al., 2010).

More recently, a motor unit number index (MUNIX) technique was developed to use different levels of voluntary surface electromyogram (EMG) signals and the compound muscle action potential (CMAP) (or maximum M-wave) to derive an index that is related to the number of motor units in a muscle (Nandedkar et al., 2004, 2010). The primary advantages of the MUNIX technique over the traditional MUNE methods lie in its minimal discomfort imposed to tested subjects as well as its quick and convenient implementation, an important feature for clinical application (Boekestein et al., 2012; Bromberg, 2013).

When the MUNIX concept was firstly defined, the main application was to evaluate motor neuron loss and disease progression in amyotrophic lateral sclerosis (ALS) (Nandedkar et al., 2004, 2010, 2011; Ahn et al., 2010; Neuwirth et al., 2010; Furtula et al., 2013). Later, the MUNIX measurement has also been applied in other disorders such as prior polio (Sandberg et al., 2011), hemiparetic stroke (Li et al., 2011b, 2014), incomplete SCI (Li et al., 2012a), and in aging and other studies as well (Li et al., 2011a; Neuwirth et al., 2011a; Drey et al., 2013, 2014a,b; Kaya et al., 2013, 2014; Ahn et al., 2015). For multifunctional muscles which can exert force on different directions about their respective joints, we have demonstrated the voluntary contraction direction dependence of MUNIX for both healthy control and ALS patients (Zhou et al., 2012, 2014). Such direction dependence is largely related to the fact that MUNIX is estimated from CMAP and voluntary surface EMG, with the former measuring the contribution of all the motor units and the latter primarily measuring the contribution of a subpopulation of motor units specifically for the voluntary contraction task (or direction) (Zhou et al., 2011). Because of this, a concept of multidimensional MUNIX (MD-MUNIX) has been proposed which utilizes voluntary surface EMG in different directions of contraction at once for the calculation of MUNIX (Zhou et al., 2014). Such an approach may result in more consistent MUNIX estimates than those solely derived from one direction of voluntary contraction.

Hand has dexterous specialties involving different fingers and the thumb (Takahashi et al., 2005). The impairment or loss of hand function (such as grasping movements and other skilled motor tasks) is among the most important factors restricting the independence of individuals with SCI. Clinical studies have shown altered finger force generation after SCI (Kim et al., 2009). In this study, the MD-MUNIX measurement was applied for examination of three multifunctional hand muscles in individuals with incomplete cervical SCI. We found that when compared with the neurologically intact subjects, the MD-MUNIX was significantly reduced for each of the examined muscles of the SCI subjects, with a concurrent enlargement of multidimensional motor unit size index (MUSIX) (MD-MUSIX). The findings of the study provide useful data on hand muscle or motor unit changes after SCI, which is important for understanding the complicated neuromuscular mechanisms underlying SCI induced hand weakness and other motor impairment. This in turn will serve to facilitate or guide development of appropriate interventions for hand function recovery.

## Methods

### Subjects

Twelve subjects (2 female and 10 male, age 49 ± 10 years) with incomplete SCI at levels of C2–C7 and American Spinal Injury Association (ASIA) impairment levels of C and D, and 12 healthy control subjects (6 male and 6 female, age 40 ± 15 years) participated in this study. All the SCI subjects were recruited through the Clinical Neuroscience Research Registry at the Rehabilitation Institute of Chicago (Chicago, IL, USA). Clinical assessment and screening examination were performed to determine the eligibility for each SCI subject. The study was approved by the Institutional Review Board of Northwestern University (Chicago, IL, USA). All the SCI and control subjects gave their written consent before any assessment or experiment procedures.

### Experiment

The experiments were performed on the first dorsal interosseous (FDI) muscle, the thenar muscle group and the hypothenar muscle group bilaterally for all the healthy control subjects. The experiments were performed bilaterally on the thenar muscles for all the SCI subjects and on the FDI and the hypothenar muscles for 8 SCI subjects. For the other 4 SCI subjects, the experiments were only performed on left side for the FDI and the hypothenar muscles. The subjects were seated in a chair with the examined forearm placed in a natural, resting position on a height-adjustable table. Each subject was instructed to fully relax at the shoulder, elbow and wrist. The laboratory maintained a constant temperature (approximately 295 K) during all the experiments.

For each examined muscle, the experiment procedures are similar to a previous study (Li et al., 2014), as briefly described below. Prior to the experiment, the skin over the ulnar and medial aspects of the wrist, the thumb, the index and little fingers, and the back of the hand were lightly abraded and cleaned with rubbing alcohol pads. Conductive gel was carefully applied to reduce skin-electrode impedance. A Sierra Wave EMG system (Cadwell Lab Inc, Kennewick, WA) was used in this study, with electrode placement following the standard motor conduction studies (Kimura, 2013). A pair of 10-mm silver/silver chloride disk surface electrodes was used to record electrical activity from the FDI, thenar and hypothenar muscles, respectively. The active surface electrode was placed on each examined muscle with the reference surface electrode positioned over the second metacarpophalangeal joint for the FDI muscle, on the metacarpal phalangeal for the thenar muscles, and on the distal phalanx of the little finger for the hypothenar muscles, respectively. An adhesive ground electrode was placed on the dorsum of the hand between the stimulus and recording sites. All the surface electrodes were further reinforced with surgical tapes to reduce movement during the recording.

For each muscle the CMAP was first recorded. A remote handheld stimulator was used to generate stimuli through a cathode (a 10 mm sliver/silver chloride pole). The duration of each single pulse stimulus was 200 μs. The M wave was evoked with a cathode placed 2 cm proximal to the wrist crease over the ulnar nerve for the FDI or hypothenar muscles, and over the median nerve for the thenar muscles. The stimulation intensity started at 15 mA, increasing approximately 20% above that until reaching the intensity eliciting the maximal response. The stimulation electrode placement was optimized by testing several different locations to confirm there was no enlargement in the M wave amplitude with further increased stimulation intensities.

After CMAP recording, the voluntary surface EMG signals were then recorded from the examined muscle with all the electrodes maintained at the same positions. For each muscle, the subject was instructed to generate isometric contraction at 4–6 different levels in a single trial, defined by the examiner offering resistance to the tested muscle. Each contraction level lasted for at least two seconds and the different contraction levels represented minimal to maximal effort. Each tested muscle performed isometric contraction in two different directions with three trials being recorded for each direction. More specifically, each subject performed index finger abduction or flexion for the FDI muscle, thumb abduction or flexion for the thenar muscles, and little finger abduction or flexion for the hypothenar muscles. Substantial rest was allowed during the experiment to avoid muscle fatigue. The sampling frequency was 6.4 kHz for CMAP and 32 kHz for voluntary surface EMG, with a band pass filter setting at 3 Hz–2 kHz and 10 Hz–10 kHz, respectively. A notch filter was applied to voluntary surface EMG recording to remove power line interference. All signals were stored to the clinical EMG machine and then transferred to a PC for offline processing.

### Data Analysis

#### MUNIX Computation

For each examined muscle, the CMAP and different levels of surface EMG interference pattern (SIP) were used to compute the MUNIX. Detailed MUNIX derivation can be found in previous studies (Nandedkar et al., 2004, 2010). In brief, the area (A_m_) and power (P_m_) of CMAP (calculated from the first negative phase), and the area (A_s_) and power (P_s_) of each level of SIP EMG (for 1 s) were first calculated. These values were used to compute the “ideal case motor unit count (ICMUC)”:

(1) $$ICMUC=\frac{P_{m}A_{s}}{A_{m}P_{s}}$$

The relation between the ICMUC and SIP area was modeled as below:

(2) $$ICMUC=\beta {\left(A_{s}\right)}^{\alpha }$$

Note that a linear regression between logarithms of ICMUC and SIP area can be used to estimate β and α. The MUNIX was defined as the ICMUC value when the SIP area equaled 20 mVms, i.e., MUNIX = β(20)^α^.

In MUNIX calculation, very low-amplitude voluntary surface EMG may result in very high ICMUC values. To reduce this artifact, three criteria were imposed to accept an SIP epoch: (1) A*_s_* > 20 mVms; (2) ICMUC < 100; and (3) A*_s_*/A*_m_* > 1 (Nandedkar et al., 2010). In addition, only those CMAPs whose amplitude is greater than 0.5 mV were accepted for the MUNIX analysis.

With MUNIX values available, a complementary measurement, motor unit size index (MUSIX), can be obtained by dividing MUNIX into the CMAP amplitude (Nandedkar et al., 2010):

(3) $$MUSIX=\frac{CMAP}{MUNIX}$$

The MUSIX of a muscle is measured in volts, which reflects the average amplitude of the individual surface MUAPs.

#### MD-MUNIX Computation

One primary feature of this study was to combine voluntary surface EMG signals from different directions of muscle contraction to calculate the MUNIX, which is defined as multidimensional MUNIX (MD-MUNIX). By contrast, previous studies routinely relied on one direction of voluntary muscle contraction for MUNIX calculation. To obtain MD-MUNIX, the SIP EMG signals from different directions of muscle contraction were used to calculate ICMUC (Equation 1). All the resultant data points were used to perform the linear regression between the logarithms of ICMUC and the SIP area to determine β and α (Equation 2). Following the MUNIX definition, the MD-MUNIX was also calculated as the ICMUC value when the SIP area was 20 mVms, i.e., MD-MUNIX = β(20)^α^. Note that β and α would be different from those derived using SIPs solely from one direction. With MD-MUNIX values available, the multidimensional MUSIX (MD-MUSIX) can be obtained by dividing MD-MUNIX into the CMAP amplitude.

### Statistical Analysis

We measured the CMAP, MUNIX and MUSIX at each contraction direction, MD-MUNIX and MD-MUSIX values in partially paralyzed FDI, thenar and hypothenar muscles of SCI subjects and in neurologically intact muscles of healthy control subjects. All the results were presented in the form of mean ± standard deviation. Student *t* test was performed to compare the difference in these parameters between SCI and control groups for each muscle. To evaluate motor unit loss for different muscles after SCI, normalized MD-MUNIX decrease, defined as MD-MUNIX*_SCI_* divided by the mean MD-MUNIX_control_, was calculated for each muscle. Within subject group one way analysis of variance (ANOVA) was applied to compare different muscles. The Bonferroni correction was used in pairwise comparison in the *post hoc* test. Significant level was determined as *p* < 0.05 for all the statistical analyses.

## Results

**Figure 1:**
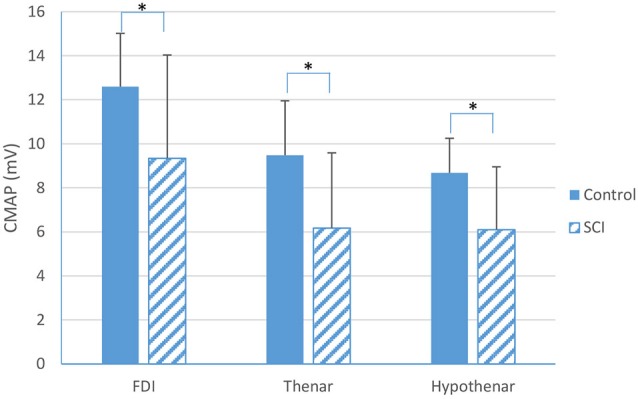
A comparison in Compound Muscle Action Potential (CMAP) between SCI and neurologically intact subjects for the FDI, thenar and hypothenar muscles (mean ± standard deviation, *indicates *p* < 0.05).

Recordings for the CMAP and voluntary surface EMG signals at different levels of contraction in two directions were obtained from the partially paralyzed FDI, thenar and hypothenar muscles and from the neurologically intact muscles. We observed a significant decrease in CMAP for all the three partially paralyzed muscles, when compared with the healthy control muscles (Figure 1). Across all subjects, the CMAP amplitude was 9.3 ± 4.7 mV for the partially paralyzed FDI muscle and 12.6 ± 2.4 mV for the neurologically intact FDI muscle (*p* < 0.01); 6.2 ± 3.4 mV for the partially paralyzed thenar muscles and 9.5 ± 2.5 mV for the neurologically intact thenar muscles (*p* < 0.001); and 6.1 ± 2.8 mV for the partially paralyzed hypothenar muscles and 8.7 ± 1.5 mV for the neurologically intact hypothenar muscles (*p* < 0.001), respectively.

Table 1 summarizes the MUNIX and MUSIX values calculated from each of the two different voluntary muscle contraction directions (i.e., flexion or abduction) for the three examined muscles of the SCI and healthy control subjects. It was observed that the MUNIX derived from the flexion mode was significantly larger than that from the abduction mode for the FDI and thenar muscles of the healthy control subjects. This resulted in significantly smaller MUSIX in the flexion mode than that in the abduction mode for the two muscles. However, there was no significant difference in MUNIX or MUSIX values between the two directions for the hypothenar muscles of the healthy control subjects and for all the three examined muscles of the SCI subjects.

**Table 1:** **Direction dependence of MUNIX and MUSIX in FDI, thenar and hypothenar muscles of healthy control and SCI subjects; the mean value from all the examined muscles is presented**.

	Muscles		
	FDI	Thenar	Hypothenar
**MUNIX**
Control	211(Abd) < 246(Flex) *	149(Abd) < 185(Flex) *	147(Abd) ≈ 151(Flex)
SCI	139(Abd) ≈ 136(Flex)	100(Abd) ≈ 102(Flex)	97(Abd) ≈ 94(Flex)
**MUSIX (μV)**	
Control	61.7(Abd) > 52.3(Flex) *	63.9(Abd) > 51.5(Flex) *	61.1(Abd) ≈ 60.7(Flex)
SCI	75.7(Abd) ≈ 78.3(Flex)	85.2(Abd) > 74.7(Flex)	75.5(Abd) ≈ 70.9(Flex)

Abd: abduction; Flex: Flexion. The asterisk (*) indicates a significant difference between two directions (p < 0.05).

**Figure 2:**
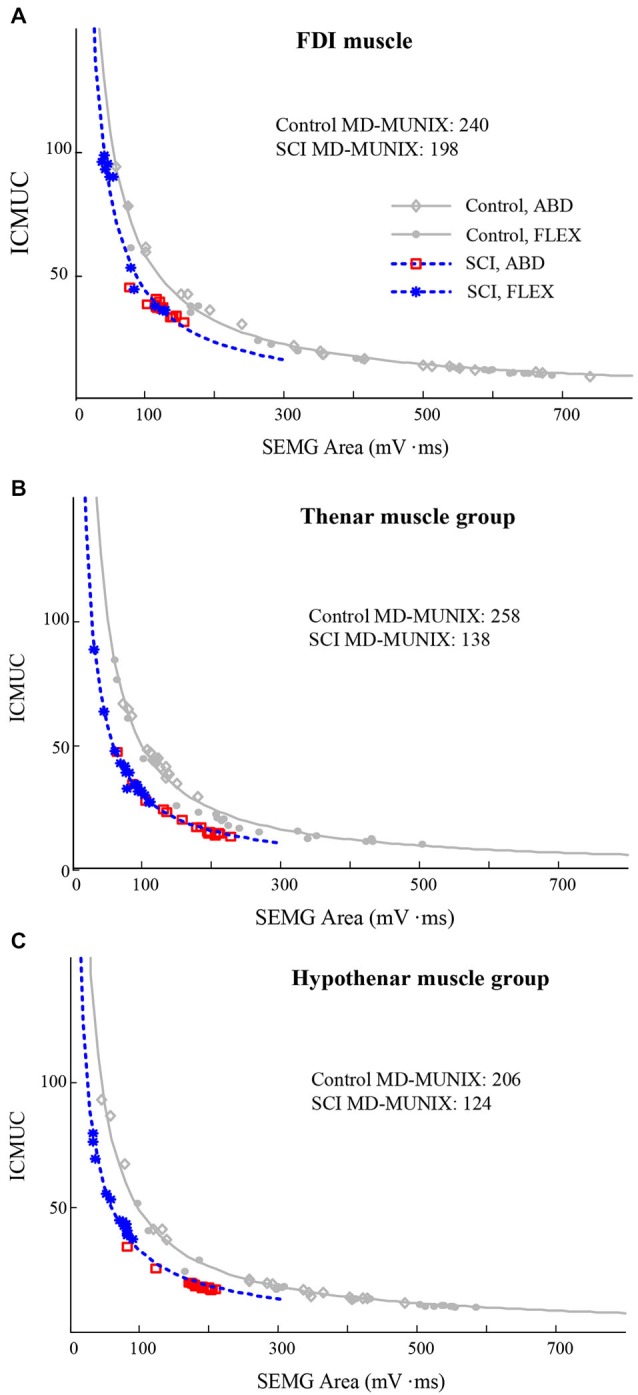
**Examples of MD-MUNIX calculation in representative SCI and healthy control subjects**. The MD-MUNIX was derived using the CMAP and the voluntary surface EMG in both abduction (ABD) and flexion (FLEX) modes for the curve fitting. **(A)** the FDI muscle; **(B)** the thenar muscles; **(C)** the hypothenar muscles.

**Figure 3:**
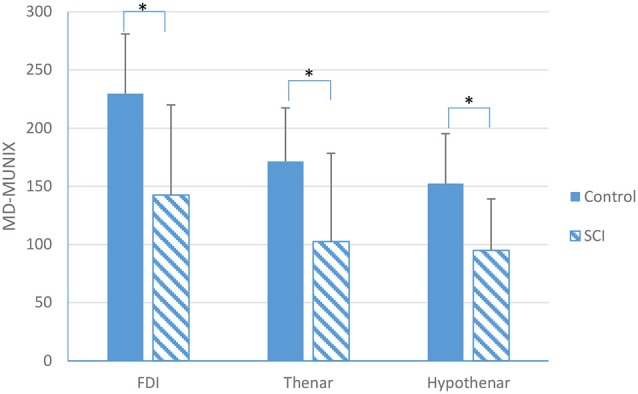
A comparison in MD-MUNIX between SCI and neurologically intact subjects for the FDI, thenar and hypothenar muscles (mean ± standard deviation, *indicates *p* < 0.05).

Figure 2 shows an example of MD-MUNIX calculation for each of the examined muscles and a comparison between representative SCI and healthy control subjects, which considered surface EMG signals from different directions of muscle contraction at a time. The values of MD-MUNIX, averaged from all the subjects, are summarized in Figure 3. A significant difference in MD-MUNIX was found between partially paralyzed and neurologically intact muscles. This was true for all the three examined muscles. Specifically, the MD-MUNIX was 143 ± 73 for the partially paralyzed FDI muscle and 230 ± 51 for the neurologically intact FDI muscle (*p* < 0.001). The MD-MUNIX was 103 ± 76 for the partially paralyzed thenar muscles and 171 ± 46 for the neurologically intact thenar muscles (*p* < 0.001). Similarly, the MD-MUNIX of the hypothenar muscles was 95 ± 44 for the SCI subjects, significantly smaller than 152 ± 43 for the healthy control subjects (*p* < 0.001).

Using one way ANOVA to compare MD-MUNIX of the three examined hand muscles within the control group, there was a significant difference among muscles, with *post hoc* test showing that the FDI’s MD-MUNIX (230 ± 51) was significantly larger than that of the thenar (171 ± 46) and hypothenar (152 ± 43) muscles (*p* < 0.001). However, for the normalized MD-MUNIX decrease (or the ratio between MD-MUNIX*_SCI_* and MD-MUNIX_control_), one way ANOVA did not reveal any significant difference among the three examined hand muscles.

**Figure 4:**
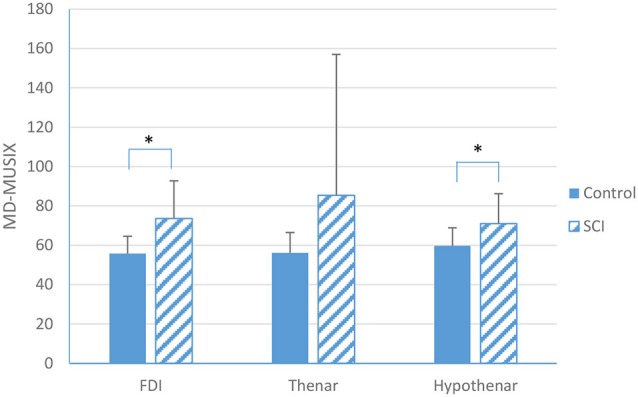
A comparison in MD-MUSIX between SCI and neurologically intact subjects for the FDI, thenar and hypothenar muscles (mean ± standard deviation, *indicates *p* < 0.05).

A summary of MD-MUSIX comparison between SCI and healthy control subjects is shown in Figure 4. It can be observed that the MD-MUSIX of the partially paralyzed FDI (73.6 ± 19.1 μV) and hypothenar (71.0 ± 15.3 μV) muscles was significantly larger than that of the neurologically intact FDI (55.8 ± 8.7 μV, *p* < 0.001) and hypothenar (59.7 ± 9.2 μV, *p* < 0.01) muscles. For the thenar muscle, the MD-MUSIX of the SCI subjects (85.4 ± 71.6 μV) was marginally larger than the healthy control subjects (56.2 ± 10.3 μV) (*p* = 0.06).

## Discussion

In this study, we used a novel concept of multidimensional motor unit indices (MD-MUNIX and MD-MUSIX) to examine partially paralyzed hand muscles of incomplete cervical SCI subjects. The MD-MUNIX combines voluntary muscle activation information in different directions and serves to address a practical question of MUNIX calculation in multifunctional muscles. This question is important considering that many muscles can produce multiple direction forces about their respective joints.

The CMAP, MD-MUNIX and MD-MUSIX were compared between partially paralyzed hand muscles of SCI subjects and neurologically intact muscles of healthy control subjects. Our results showed that for each of the examined hand muscles, the amplitude of CMAP was significantly lower in SCI subjects than that in healthy control subjects. This finding is in line with previous observations from individuals with SCI (Kirshblum et al., 2001; Van De Meent et al., 2010; James et al., 2011; Riley et al., 2011). Our results also revealed significantly lower MD-MUNIX in hand muscles of the SCI subjects compared to the neurologically intact subjects. This was true for all the three examined muscles (FDI, thenar and hypothenar). The finding is consistent to previous MUNE studies in SCI which demonstrated variable levels of motor unit loss in paralyzed muscles (Yang et al., 1990; Xiong et al., 2010; James et al., 2011). In a previous study using conventional MUNIX calculation (that relied on one direction of voluntary muscle contraction), significantly reduced MUNIX was found in the FDI muscle of the tested cervical SCI subjects compared to the healthy control subjects (Li et al., 2012a). The consistent finding from the MD-MUNIX measurement of the FDI muscle as demonstrated in the current study provides further evidence of muscle structural changes (motor unit loss/inactivation, muscle fiber denervation/reinnervation or atrophy, etc.) following the spinal injury. In addition to the FDI muscle, significantly reduced MD-MUNIX was also observed from the thenar and hypothenar muscles of the SCI subjects, providing further evidence of such changes.

A significant difference in MD-MUNIX value was observed for the three examined muscles of the healthy control subjects (one way ANOVA, *p* < 0.05) in a sequence of FDI > thenar > hypothenar muscles. In a previous multi-center study with healthy subjects (less than 60 years), a higher MUNIX range was also reported for the abductor pollicis brevis muscle compared to the abductor digiti minimi muscle (Neuwirth et al., 2011b). It should be noted that both MUNIX and MD-MUNIX represent an index of the motor unit numbers in a muscle. The MUNIX or MD-MUNIX comparison of different muscles may not necessarily be parallel or related to the comparison of actual motor unit counts in these muscles (as suggested by various MUNE methods). Thus, the focus of the MUNIX (or MD-MUNIX) study should be the MUNIX (or MD-MUNIX) changes in the same muscle of different groups (e.g., between neurologically intact and diseased muscles, or tracking muscle changes in a longitudinal study).

Given the significant MD-MUNIX reduction in SCI compared with the healthy control subjects, we also calculated the normalized MD-MUNIX decrease (i.e., the ratio of SCI’s MD-MUNIX to the mean MD-MUNIX of the healthy control subjects) for each of the three examined muscles. It was found that there was no significant difference among the FDI, thenar and hypothenar muscles in the normalized MD-MUNIX decrease. These findings suggest that hand muscles or motor units might be universally affected after cervical SCI, which can jeopardize force output and other functions of the hand.

We also calculated the conventional MUNIX and MUSIX for each examined muscle using one direction (i.e., either abduction or flexion) of voluntary muscle contraction (Table 1). Our results showed that for the neurologically intact FDI and thenar muscles, the MUNIX derived from the two different directions of voluntary muscle contraction was significantly different. For example, the MUNIX derived from the FDI flexion mode was significantly larger than that from the abduction mode. This finding was also reported in a previous study (Zhou et al., 2012). The same pattern was also observed for the thenar muscles in the current study. By contrast, such a difference appeared not significant for the tested SCI subjects, which was true for all the three examined muscles. This implies that the direction dependence of MUNIX calculation might be obscured with neurological injuries. In a previous MUNIX study of ALS subjects (Zhou et al., 2014), it was observed that the MUNIX of one ALS subject’s FDI muscle derived from the index finger abduction mode was much higher than that from the flexion mode. This was different from what observed in the neurologically intact subjects and in most of the tested ALS subjects. Such an observation may arise from the subject’s difficulty in voluntarily activating the FDI muscle during index finger abduction (compared with index finger flexion), as a result of selective motor neuron or motor unit pool impairment following the disease (Zhou et al., 2014). In the current study, the lack of significant difference in MUNIX values calculated from muscle contraction in each different direction could also be possibly due to selective impairment of the multifunctional muscles as a result of SCI, which may obscure the direction dependence of MUNIX calculation. On the other hand, this also demonstrates the importance of applying MD-MUNIX measurement for a more reliable or comprehensive assessment of the multifunctional muscles.

In the current study, in addition to significantly reduced MD-MUNIX for all the three examined muscles in SCI, significantly larger MD-MUSIX values were observed from the FDI and hypothenar muscles of the SCI subjects when compared to the healthy controls. For the thenar muscles, the MD-MUSIX value was also larger for the SCI group at a close-to-significance level. Our previous simulation study (Li et al., 2012b) using motor neuron pool and surface EMG models has revealed that MUNIX reduction may be either from actual motor unit loss or/and from muscle fiber atrophy. In such a case, MUSIX would be a good accompanying parameter to provide more definitive information. In the present study, significant reductions of the MD-MUNIX together with the enlargement of MD-MUSIX in the SCI subjects provide supportive data suggesting motor unit loss and muscle fiber reinnervation as a secondary evidence of this loss in the partially paralyzed hand muscles after cervical SCI.

In summary, this study presents an examination of three hand muscles in subjects with incomplete cervical SCI, using novel multidimensional motor unit indices (MD-MUNIX and MD-MUSIX). The results indicate that the SCI subjects had significantly smaller MD-MUNIX in all the three investigated muscles, compared to those derived from the healthy control subjects. The FDI and hypothenar muscles demonstrated significantly larger MD-MUSIX values in the SCI subjects than those of the healthy control subjects, whereas the thenar muscle demonstrated marginally significant MD-MUSIX enlargement. The findings from the MD-MUNIX analyses provide an evidence of motor unit loss in hand muscles of individuals with cervical SCI, which may contribute to weakness and other hand function deterioration.

## Conflict of Interest Statement

The authors declare that the research was conducted in the absence of any commercial or financial relationships that could be construed as a potential conflict of interest.
